# A bottom‐up framework for nurses' protocol‐based care decision‐making

**DOI:** 10.1002/nop2.2232

**Published:** 2024-09-15

**Authors:** Monica Vázquez‐Calatayud, María J. Pumar‐Méndez, Cristina Oroviogoicoechea

**Affiliations:** ^1^ Area of Nursing Professional Development and Research Clínica Universidad de Navarra Pamplona Spain; ^2^ Innovation for a Person‐Centred Care Research Group (ICCP‐UNAV) University of Navarra Pamplona Spain; ^3^ Navarra Institute for Health Research (IdiSNA) Pamplona Spain; ^4^ Department of Health Sciences Public University of Navarre (UPNA) Pamplona Spain; ^5^ Capacity Building for Health Promotion Research Group (CreaP) Public University of Navarre (UPNA) Pamplona Spain; ^6^ Area of Nursing Innovation Clínica Universidad de Navarra Pamplona Spain

**Keywords:** decision‐making, framework, nursing, nursing education, protocol‐based care

## Abstract

**Aim:**

To develop a conceptual framework for nurses' protocol‐based care decision‐making.

**Design:**

Miles & Huberman's bottom‐up approach to developing conceptual frameworks was followed, using data collected from a multiple embedded case study examining protocol‐based decision‐making by nurses in three hospital wards within a university hospital in northern Spain.

**Methods:**

The qualitative data from the case study, obtained through documentary analysis, observations, and interviews, underwent a secondary analysis consisting of four steps: data reduction, data display, comparison, and drawing conclusions.

**Results:**

The framework for protocol‐based care decision‐making comprises four components: (1) protocol‐based care, as a balance between standardisation and individualised care, (2) the process, (3) the context, and (4) the elements of protocol‐based care decision‐making. These components and their relationship as a context‐dependent, linear, variable and multifactorial process, directly influenced by the perception of risk, are described and illustrated.

**Conclusions:**

This study provides a rigorous bottom‐up framework for nurses' protocol‐based care decision‐making. The framework could be a valuable resource for managers, clinical nurses, educators, and researchers to guide and evaluate nurses' decision‐making, leading to improved care quality and reduced variability in clinical practice. Furthermore, the framework lays a foundation for further research and practical applications.

**Impact:**

This study addressed the problem of understanding nurses' protocol‐based care decision‐making and the need for a specific conceptual framework. The main findings of the study contribute to the development of a rigorous bottom‐up framework comprising four components of protocol‐based care decision‐making. The framework has the potential to improve care quality, reduce variability, enhance patient safety, and increase healthcare efficiency by guiding nurses' decision‐making in various healthcare settings.

**No Patient or Public Contribution:**

Patient or public contribution was not applicable since the study focused on nurses' decision making.

## 1 INTRODUCTION

Unwarranted variability in clinical practice remains a pressing quality issue in healthcare today (Atsma et al., 2020). To address this challenge, protocol‐based care (PBC) has emerged as a strategy that balances care standardisation and individualisation by using tools for standardisation in a reasoned way (Vázquez‐Calatayud et al., 2020). This approach requires healthcare professionals to gather, interpret, and evaluate patient information to select an individualised course of action that meets evidence‐based standards while prioritising patients' well‐being (Vázquez‐Calatayud et al., 2020).

To promote successful implementation of PBC in clinical practice, it is essential to understand the PBC decision‐making process and develop a specific conceptual framework (Vázquez‐Calatayud et al., 2020; Fawcett, 2017; Jozkowski, 2017). This article aims to answer the research question of how the key components and influences that shape nurses' decision‐making in PBC can be structured into a comprehensive conceptual framework. The resultant framework is based on a secondary analysis of data generated from a multiple embedded case study investigating PBC decision‐making by nurses in three hospital wards within a university hospital in northern Spain (Vázquez‐Calatayud et al., 2020).

## 2 BACKGROUND

The ambiguity surrounding the definition of PBC complicates efforts to conceptualise PBC decision‐making. Ilott et al. (2006) conducted a conceptual analysis of PBC and concluded that PBC involves staff following and retaining responsibility for the appropriate use of protocols, care pathways, and clinical guidelines that represent codified rules aiming at improving healthcare standardisation and outcomes. Subsequently, Rycroft‐Malone et al. (2010) referred to PBC as the reasoned use of tools for the standardisation of care to achieve the balance between the standardisation and individualisation of care, based on self‐report and observational data.

These two portrayals offer different approaches to PBC, with Ilott et al. (2006) emphasising standardisation over individualisation of care, while Rycroft‐Malone et al. (2010) focus on balancing both practices. However, neither Ilott et al. (2006) nor Rycroft‐Malone et al. (2009, 2010) clarify another fundamental aspect of PBC: what is meant by the appropriate use of tools for care standardisation.

PBC Decision‐Making involves selecting a course of action that makes appropriate use of tools for the standardisation of care on the specific requirements of each situation, which may involve modifying or adhering to their use (Vázquez‐Calatayud et al., 2020). This process is critical for the successful implementation of PBC and reducing variability in clinical practice. However, to ensure the successful implementation of PBC, it is important to have a comprehensive understanding of the process of PBC decision‐making and to develop a specific conceptual framework that guides further research and practical application in clinical settings (Vázquez‐Calatayud et al., 2020; Fawcett, 2017; Jozkowski, 2017).

To date, no framework has been developed specifically for PBC decision‐making, and existing frameworks tend to focus on clinical decision‐making as a whole and lack empirical grounding (Ballard et al., 2016; Nibbelink & Brewer, 2018; O'Connor, et al., 2023). While PBC decision‐making shares similarities with general clinical decision‐making, such as the patient‐centred approach and evidence‐based practice, a more targeted understanding and application of PBC principles are required. Therefore, this paper aims to fill this gap by presenting a novel conceptual framework for PBC decision‐making that can assist clinical and academic educators in designing strategies that promote the successful implementation of PBC.

## 3 METHOD

To construct a framework on nurses' PBC decision‐making, the study followed the approach described by Miles and Huberman (2019), which defines a conceptual framework as a graphic or narrative representation of ‘key factors, constructs or variables and the presumed relationship among them’ (p.18). Miles and Huberman (2019) proposed two approaches for constructing such a framework, the top‐down approach, which starts from the conceptual framework and works towards collecting data to test its validity, and the bottom‐up approach, which starts from the field and works towards developing the concepts. In this study, the bottom‐up approach was adopted, following the key steps of data reduction, data display, comparison, and drawing conclusions (Miles & Huberman, 2019, p. 26).

This methodology was applied to data derived from a multiple embedded case study previously conducted by the authors. This case study aimed to describe and explain nurses' protocol‐based care (PBC) decision‐making across three hospital wards (medical, surgical, and medical‐surgical) within a university hospital in northern Spain, focusing on four protocols: blood transfusion, prevention of falls, contact isolation, and hand hygiene. Data collection included documentary analysis, non‐participant observations, participant observations, and interviews. The detailed findings of this study have been previously published elsewhere (Vázquez‐Calatayud et al., 2020).

**FIGURE 1:**
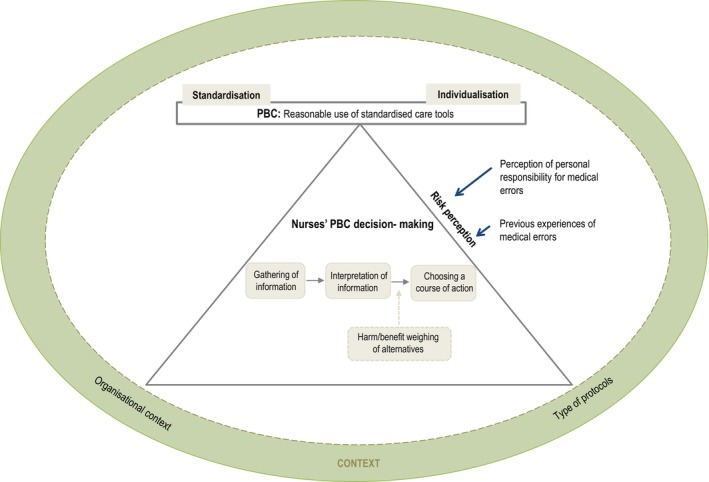
Framework on nurses' PBC decision‐making.

Thus, firstly, the extensive datasets acquired from the cited case study underwent a process of data reduction, which involved choosing relevant data and simplifying, abstracting, and transforming it into more manageable forms. Subsequently, these filtered datasets were organised and presented coherently through data display, which could include textual descriptions, diagrams, charts, or matrices. This organised presentation aided in drawing conclusions by offering a fresh perspective on the available data. Finally, emergent conclusions were meticulously scrutinised through comparison and revisiting of the original data to ensure their validity and reliability. Table 1 provided an overview of the specific strategies used in each of these steps to develop the framework based on the data obtained. Ultimately, these steps enabled the researchers to represent the constructs and their relationships within a conceptual framework (Figure 1). Files S1 and S2 provide further details on the data display matrices used in the study.

**TABLE 1:** Steps and strategies for the development of the conceptual framework.

Steps	Strategies
1. Data reduction	The qualitative data (documents, field notes and transcripts from observations and interviews) of each case were subjected to a content analysis Exhaustive readings of the dataset were carried out A system of categories to describe the units of meaning identified concerning the phenomenon under study was developed The category system was revised and refined based on the identification of common patterns in the data The categories were ordered and regrouped into broader themes to describe and explain PBC decision‐making
2. Data display	Intracase and cross‐case data display. For each case and for the three cases together, the data were tabulated in matrices (see Files S1 and S2) The matrix relates the propositions of the study, represented in the columns of the matrix, with the findings derived from the different data sources, which are included in the corresponding rows. The matrix made it possible to vertically compare the set of data relating to each of the propositions and to construct a new perspective on decision‐making in PBC in terms of the context that conditions it, its process and its nature The findings of each case, when contrasted and integrated with the findings of the other two cases, were completed and represented in a new matrix. In the columns of this matrix, the cases of the study were included together with the description of the surrounding context, while in the rows, the final propositions and subpropositions were arranged
3 and 4. Comparison and drawing conclusion	By means of the matrix that allowed cross‐case display comparison, it was possible to horizontally compare and integrate the set of findings for the cases in the light of each of the final propositions and subpropositions and to obtain a new vision of the context that conditions decision‐making in PBC, its process and nature. In this way, it was possible to represent the constructs and their relationships to each other in a conceptual framework (Figure 1)

Source: Miles and Huberman (2019), Vázquez‐Calatayud et al. (2020).

The multiple embedded case study that provided the datasets used to develop the conceptual framework was conducted by the authors of this study, meaning they were familiar with the datasets used for the secondary analysis. The first author took responsibility for applying each step of Miles & Huberman's bottom‐up approach to developing conceptual frameworks. The outcomes of this process were independently reviewed by the other two authors, who resolved any disagreements, questions, or inconsistencies in meetings involving all three researchers.

## 4 RESULTS

The results of the study present a conceptual framework for nurses' PBC decision‐making that was developed through an iterative process of data reduction, data display and comparison. The framework consists of four components: PBC, the PBC decision‐making process, context, and elements of the PBC decision‐making. These components and their relationship are represented graphically in Figure 1.

### 4.1 PBC

As illustrated in Figure 1, PBC is represented as balanced approach between standardisation and individualised care. It involves the reasoned use of care standardisation tools in response specific situations. Nurses may opt for standardised care by adhering to the prescribed tools or individualised care by modifying or omitting some steps. Thus, the optimal decision for each situation depends on achieving a balance between standardisation and individualization.

### 4.2 PBC decision‐making

PBC decision‐making is established as a core process to achieve a balance between the standardisation and individualisation of care (see Figure 1). Our study on nurses' PBC decision‐making revealed that this decision‐making involves a linear and variable process, where the number of phases and their sequence depend on the clinical situation and the type of protocol being applied. In routine clinical situations or in protocols with a high degree of risk associated with their violation, the process is limited to three phases of gathering information, interpreting information, and choosing a course of action. For example, considering the case of an oncology patient with anaemia who has been prescribed a blood transfusion to improve their haemodynamic status, the application of PBC decision‐making would involve collecting and interpreting information about the patient's close monitoring data, before, during and after transfusion, and choosing to strictly apply the protocol to avoid the risk of a fatal consequence if it has deviated.

On the other hand, in situations where there is no risk, and modification to protocols can result in greater well‐being for the patient, the process includes an additional phase of weighing the risks and benefits of the possible alternatives. For instance, the nurses in our study decided that modifying the pressure ulcer protocol and spacing postural changes beyond what was recommended by the prescription was the most beneficial course of action for a patient who was resting after a night of distress and pain.

### 4.3 Context of the PBC decision‐making

PBC decision‐making is considered context dependent. In particular, as shown in Figure 1, the type of protocol influences the way decisions are made in PBC. As explained above, the decision‐making process varies, incorporating three to four phases depending on the *type of protocol* and whether the protocol has a high or moderate degree of risk associated with its violation.

In addition, PBC decision‐making is conditioned by the *organisational context* in which it takes place, as also shown in Figure 1. The organisational climate, the style of supervision and the way of performing coaching influence the development of the PBC decision‐making process and the elements that intervene in it. In particular, certain aspects of the organisational climate were identified, such as the attitude towards protocols as well as interprofessional and intraprofessional relations, which influence professionals to seek a balance between standardisation and individualisation of care. In addition, it was found that coaching offered by an advanced practice nurse (APN) was more effective in the improvement of decision‐making processes to combine the standardisation and individualisation of care.

### 4.4 Elements of the PBC decision‐making

The PBC decision‐making process is also identified as directly influenced by the *perception of risk* as a single and central element (Figure 1). The perception of risk refers to nurses' beliefs about the possibility of exposing a patient to risk as a result of not following the protocols and their beliefs about the magnitude of the effect of that exposure. When nurses perceived risk to the patient, they prioritised safety, opting to prioritise standardisation and leave little room for individualisation. For instance, one of the nurses interviewed in the study explained that if they performed a cure or a sterile procedure, they decided to wash their hands because the procedure implied a risk of infection. However, if they administered pills to a patient, they did not wash their hands because they did not touch or do anything that would pose a risk of infection.

The direct effect of risk perception on PBC decision‐making, towards greater or less standardisation, is modulated or influenced by *previous experiences of medical errors* and *perception of personal responsibility for medical errors*, as shown in Figure 1. Previous experiences of medical errors associated with the misuse of protocols, therefore, led nurses to be more aware of the risks and the importance of applying the protocols correctly.

On the other hand, it was found that when nurses believed that the harm caused to the patient depended solely on their actions, they felt a greater responsibility for medical errors and thus a greater perception of risk. However, when they considered that the risks and damages to which patients were exposed could be generated by the actions of different professionals, nurses perceived less responsibility for them, and their risk assessment seemed to be attenuated. For example, some nurses in the study reported that they felt less aware of the negative effect of not washing hands between patients when the patient's care was dependent not only on them but also on the actions of other professionals.

## 5 DISCUSSION

To the best of our knowledge, this study introduces a novel conceptual framework for PBC decision‐making among nurses. By articulating the fundamental components of this framework, namely the linearity, variability, multifactorial nature, and contextual dependence of the decision‐making process, the study offers new insights into how nurses make PBC decisions. Additionally, the study proposes potential avenues for future research, as well as implications for managers, clinical nurses, and educators.

This study makes a significant contribution to the nursing field by presenting a conceptual framework for nurses' PBC decision‐making. This framework is particularly relevant since nursing discipline rarely uses conceptual frameworks to guide research and practice. When utilised, such frameworks often stem from other disciplines (Fawcett, 2017). Therefore, the development of a conceptual framework based on the nursing field is necessary to expand knowledge of the complex and context‐dependent phenomenon of PBC decision‐making (Miles & Huberman, 2019; Yin, 2017). The refinement of this framework is needed through its application in various contexts, leading to an accumulation of knowledge and a better understanding of the PBC decision‐making phenomenon (Yin, 2017). Furthermore, comparing this knowledge with other similar contexts can expand and generalise theories (Jones‐Hooker & Tyndall, 2023).

The conceptual framework for nurses' PBC decision‐making presented in this study can serve as a useful tool for managers to guide and evaluate nurses in their decision‐making regarding the use of protocols, which can ultimately lead to better care quality and reduced variability in clinical practice. For instance, the framework could be integrated into existing programmes such as the Best Practice Spotlight Organisations international programme (Moreno‐Casbas et al., 2019), aimed at supporting the implementation of best practice guidelines and evidence‐based practices to optimise clinical and health outcomes. This framework has the potential to transform the way nurses make decisions regarding PBC and contribute to the overall improvement of healthcare systems.

Given the context‐dependent nature of PBC decision‐making, managers should prioritise creating the necessary contextual conditions for the effective implementation of protocols to fully leverage the potential of the proposed framework. This includes fostering an organisational climate characterised by positive attitudes towards protocols, shared co‐responsibility for their development and revision, and effective intra/interprofessional collaboration. In particular, shared co‐responsibility with an advanced practice role supports better nurses' PBC decision‐making. A recent study (Vázquez‐Calatayud et al., 2022) suggests that this may be due to the APN's facilitation of self‐reflection, guidance in questioning the best evidence‐based alternatives, and encouragement of a critical attitude towards considering different options rather than acting automatically.

The proposed conceptual framework for PBC decision‐making can serve as a useful model for clinical nurses to understand and reflect on areas that require improvement in contexts consistent with those where the research has been conducted. Nurse educators can also use the framework to analyse and train nurses' decision‐making in academic and clinical settings. Two variables that modulate the PBC decision‐making process, the degree of clinical activity risk and the availability of clues for optimising patient well‐being, should be emphasised by educators (Vázquez‐Calatayud et al., 2020; Dunn et al., 2023). In clinical settings where care does not involve life‐threatening decisions, nurses should receive training on how to weigh the risks and benefits of possible alternatives, as such situations may become more frequent. To ensure effective training for nurses from different generations with varying values and needs, educators should consider using different formats such as face‐to‐face, distance learning, or simulations (Vázquez‐Calatayud et al., 2021; Hakvoort et al., 2022).

Finally, the conceptual framework also provides a solid foundation for further research and practical applications related to nurses' PBC decision‐making. The framework can serve as a basis for developing tools to measure PBC decision‐making and designing complex interventions for its effective implementation (Bauer & Kirchner, 2020; Fawcett, 2017; Skivington et al., 2021). Nursing interventions and research grounding in robust conceptual frameworks can lead to sustainable positive outcomes and the advancement of nursing discipline (Fawcett, 2017; Juniarti et al., 2019). To further refine and expand the framework, future research could incorporate Delphi studies, as suggested by McMeekin et al. (2020) in their recent scoping review on framework development.

### 5.1 Limitations

The conceptual framework presented in this paper was developed based on a single study conducted in a hospital with a particular cultural and health context. Nevertheless, it is worth noting that it was developed using a rigorous methodology for the development of conceptual frameworks. As previously mentioned, the framework was derived through an iterative process, from empirical data to theory and vice versa. Moreover, the data was generated from a robust multiple case study where the perspective of the nurses who made daily PBC decisions was investigated, so this framework corresponds to the practical reality and should be easy for nurses to understand and apply.

## 6 CONCLUSIONS

This study provides a bottom‐up framework for nurses' PBC decision‐making, developed through a rigorous methodological approach. The complexity, linearity, variability, context dependence, and multifactorial nature of this phenomenon are described and illustrated in detail. This framework can serve as valuable resource for managers, clinical nurses, educators, and researchers in guiding and evaluating nurses' decision‐making regarding the use of protocols, leading to better care quality and reduced variability in clinical practice. The framework provides a solid foundation for further research and practical applications, such as developing tools to measure PBC decision‐making and designing complex interventions for its effective implementation.

## AUTHOR CONTRIBUTIONS

**Table:** 

Criteria	Author initials
Made substantial contributions to conception and design, or acquisition of data, or analysis and interpretation of data	MV‐C; CO; MJP‐M
Involved in drafting the manuscript or revising it critically for important intellectual content	MV‐C; CO; MJP‐M
Given final approval of the version to be published. Each author should have participated sufficiently in the work to take public responsibility for appropriate portions of the content	MV‐C; CO; MJP‐M
Agreed to be accountable for all aspects of the work in ensuring that questions related to the accuracy or integrity of any of the work are appropriately investigated and resolved	MV‐C; CO; MJP‐M

## FUNDING INFORMATION

This research received funding from a grant provided by Maphre Foundation, “Ignacio Hernando de Larramendi” (2014/BIL/14/S2/048).

## ETHICS STATEMENT

The investigation was approved by the Research Ethics Committee (code 146/2014).

## Supporting information


File S1.

File S2.

## Data Availability

Data available on request from the authors.
