# Supplementary material for: A bottom‐up framework for nurses' protocol‐based care decision‐making
Source: Nurs Open. 2024 Sep 15;11(9):e2232. doi: 10.1002/nop2.2232 (PMC11403125; doi:10.1002/nop2.2232)
Supplement: Supplementary file 1 — File S1. [file NOP2-11-e2232-s002.docx]

**Supplementary File 1.** Matrix to examine the propositions in each dataset for case A

| **Source of data** | **Proposiciones** | | |
| --- | --- | --- | --- |
|  | **1. PBC decision-making depends on the organizational context and the type of protocol** | 1. **PBC decision-making is an iterative and variable process** | **3. PBC decision-making consists of multiple interrelated elements** |
| Documentary analysis | - The institutional strategy does not name PBC but values and promotes two essential aspects for it to occur: standardisation and individualisation of care. - There are means to implement and improve standardisation: the process of drawing up and disseminating protocols, working groups, material resources, training in the use of protocols, methods for evaluating professional performance, monitoring the use of protocols and annual improvement objectives for the service relating to compliance with protocols and their correct recording. - The staffing plan in its definition takes into account the individualisation of care. - The roles of the nursing professionals are described. The manager is responsible for the training and assessment of nurses, resource management and ensuring the provision of individualised care; the CNS, collaborating in the monitoring of protocols, encouraging the participation of nurses in working groups for their implementation and coaching on their use; and the nurses, providing care based on patient assessment and protocols. - There are nurses in the service with varied experience in terms of the number of years they have been in the service. | | |
| Context observations | - Medical inpatient department, where oncology patients, mostly immunosuppressed, are admitted. - The staffing and the care load allow the needs of the patients to be met and the protocols to be applied. - Organisational climate: collaborative work, open communication and a positive attitude towards protocols. - The department has mechanisms in place to promote standardisation and/or individualisation: monitoring of protocol compliance by the supervisor and CNS, coaching on how to apply protocols by the CNS , training in protocol application, dissemination of information on the results of protocol application, high staff participation in the development of department-specific protocols, and a patient-adaptable care recording and planning system. | | |
| Participant observations | - PBC occurs with a high frequency in most protocols: ≥ 90% in protocols with high and moderate associated risk; and 30-90% in protocols with low risk, with its application in infected or immunocompromised patients standing out. Standardisation prevails over individualisation, with a greater margin for individualisation in protocols with flexibility and moderate risk. | | |
|  | - Coaching on the use of protocols helps them understand the importance of standardising or individualising care in each situation. | - The cost-benefit weighing phase of alternatives is included in the use of protocols with moderate flexibility and associated risk. | - Risk perception is involved in PBC decision-making. |
| Interviews | - Coaching and monitoring of the use of protocols are valued by the nurses to favour the application of protocols. - The organisational climate is marked by collaborative work and a positive attitude towards protocols. - The way of disseminating information is not always perceived as adequate to achieve receptiveness of the nurses. | | |
|  | - Coaching on the use of protocols improves the development of the information interpretation and cost-benefit weighing phases of alternatives. . | - The process is linear and variable, sometimes including 3 phases, sometimes 4. - The first two phases are considered the most relevant. - The phase of weighing the cost-benefit of alternatives is incorporated when patient safety and well-being make it advisable, in the use of protocols with flexibility and moderate associated risk. | - Risk perception is involved in PBC decision-making. Risk is conceived as the possibility of the occurrence of immediate harm with greater or lesser severity. Risk perception is influenced by previous experiences of medical errors and the perception of personal responsibility for those errors. |

CNS: Clinical Nurse Specialist; PBC: Protocol Based Care.
