# Supplementary material for: A bottom‐up framework for nurses' protocol‐based care decision‐making
Source: Nurs Open. 2024 Sep 15;11(9):e2232. doi: 10.1002/nop2.2232 (PMC11403125; doi:10.1002/nop2.2232)
Supplement: Supplementary file 2 — File S2. [file NOP2-11-e2232-s001.docx]

**Supplementary File 2.** Matrix to examine the propositions of the study in each set of case study outcomes

CNS: Clinical Nurse Specialist; PBC: Protocol Based Care.

|  | | **Case A** | **Case B** | **Caso C** |
| --- | --- | --- | --- | --- |
| **Proposicions y sub-proposicions** | **Case study** | Medical hospitalisation service, which belongs to an institution that, without having PBC as a working approach, values and promotes key aspects of PBC.  PBC is applied across the board in all protocols, with standardisation taking precedence; individualisation only occurs in protocols with flexibility and moderate risk; in protocols with low risk, it is restricted to infected or immunosuppressed patients, who predominate in the department. Punctual and limited monitoring of compliance with protocols, highlighting the coaching exercised by the CNS on their use. The organisational climate is favoured by open communication, collaborative work and a positive attitude towards protocols. | Surgical hospital service marked by the strategy of the institution to which it belongs, which, without being the focus of its work, promotes essential aspects of PBC.  PBC occurs more frequently in protocols with high and moderate associated risk than in protocols with low risk. Standardisation prevails over individualisation. Although the attitude towards some protocols is sometimes negative, PBC is promoted by thorough monitoring of protocol compliance by the whole team and coaching by the supervisor. There is open communication and collaboration between the nurses in the service, who are highly experienced, which contrasts with the opacity and lack of collaboration with the doctors. | Medical-surgical hospital service determined by a strategic plan that highlights and promotes key aspects of PBC.  It is characterised by a high care load, a shortage of staff and the care of patients with the greatest life-threatening conditions. It is distinguished by the fact that PBC hardly ever occurs. PBC only occurs in situations involving severe risk, with standardisation prevailing in all instances, with no room for individualisation. Barriers to the application of PBC are the high burden of care, disorganisation, peer pressure and negative attitudes towards protocols. |
| **1. Decision-making depends on organisational context and type of protocols.**  1.1. Organisational culture influences decision-making in PBC.  1.2. Coaching on the use of protocols influences decision-making in PBC.  1.3. Activity type influences PBC decision-making. . | | There are two aspects of the context that condition decision-making in PBC: the type of protocols and the coaching on their use that affect the decision-making process. | There are two contextual aspects that condition decision-making in PBC: the type of protocols that affect the process and the type of activity that influences its constituent elements. | There is only one aspect of the context: the type of patient that affects the elements that constitute decision-making in PBC. |
| **2. Decision-making in the PBC is a linear and variable process.** | | The decision-making process in PBC comprises up to four phases: gathering and interpreting information, weighing the cost-benefit of alternatives and selecting a course of action, which follow one after the other in a linear fashion. The first two phases stand out, while the phase of weighing the cost-benefit of alternatives is only developed in specific instances and decisions regarding the use of protocols with flexibility and a moderate degree of risk, being a variable process. | The process is variable, sometimes comprising three phases, sometimes four, which are sequenced in a linear fashion. The phase of weighing the cost-benefit of alternatives is incorporated in the use of flexible protocols with moderate associated risk, when the safety and well-being of the patient make it advisable to adapt them. The phases of data collection and interpretation are of particular relevance. | The decision-making process in the PBC manifests itself as the linear and invariable achievement of three phases, the most important of which is the collection and interpretation of information. |
| **3. PBC decision-making consists of multiple interrelated elements**  3.1. Perception of risk is directly involved in PBC decision-making.  3.2. Perceived knowledge of the patient is directly involved in PBC decision-making.  3.3. Experience is directly involved in PBC decision making.  3.4. Awareness of the importance of applying protocols correctly, the experiences associated with their use and the perception of personal responsibility for these errors are indirectly involved in PBC decision-making by influencing the perception of risk. | | Risk perception is directly involved in PBC decision-making, where risk is understood as the possibility of the occurrence of harm to the patient, with greater or lesser severity, derived from the use of protocols. Risk perception is influenced by previous experience of medical errors and personal perception of those errors. | Risk perception is directly involved in PBC decision making, with risk being conceived as the possibility of the occurrence of more or less severe harm to the patient. This element is only influenced by previous experience of medical errors.  Experience is identified as a new element directly involved in PBC decision-making. | Risk perception is directly involved in PBC decision making, with risk being conceived as the possibility of the occurrence of more or less severe harm to the patient. This element is only influenced by previous experience of medical errors.  Experience is identified as a new element directly involved in PBC decision-making. |
